# The role of women in imaging: the evidence

**DOI:** 10.1093/bjr/tqaf282

**Published:** 2025-11-19

**Authors:** Fiona J Gilbert

**Affiliations:** Department of Radiology, Clinical School, University of Cambridge, Cambridge CB2 0QQ, United Kingdom

**Keywords:** women, leadership, EDI, career, affirmative action, radiology

## Abstract

Since the discovery of x-rays, the role of women in imaging has become increasingly important. Reflecting changes in the roles of women in society, and the increasing acceptance of women’s effective and complementary contribution to the workforce, the number of women working in imaging has increased in the past few decades. This is due in part to the change from male-only medical schools early last century to equal numbers of male and female medical students in many countries by the 1970s. Radiology is an attractive career option for women, and over the past 2 decades the proportion of women in the field has increased to approximately 41%. This has resulted in an increase in the number of women in leadership roles. This article examines the evidence for women in various roles, explores why having women in positions of influence is important, and looks at trends in the data. The recent move away from Equality, Diversity, and Inclusion (EDI) initiatives is disappointing, as these measures helped increase the number of women in senior roles. It is clear there are many opportunities for professional women to step up and we should encourage and support them to do so.

## Introduction

Despite equal numbers of women in most medical schools worldwide, there is still not parity at consultant level in radiology. Radiology welcomes female applicants and does not require a higher degree for entry. Training can also be undertaken part-time. While on-call can be challenging and onerous, many departments find solutions to ensure that weekend, evening, and overnight duties are sustainable. Female role models are important to attract the next generation of women into radiology. This article examines the various ways women are recognized and how the numbers of women in senior positions have gradually increased over recent years.

## What is the evidence for women in leadership roles in imaging?

Marie Curie immediately springs to mind when one starts to consider the role of women in imaging and oncology. This physicist and chemist of the 20th century worked tirelessly to bring her discovery of radium and polonium to the treatment of cancer, and she has been rightly recognized. However, the contribution of Dorothy Franklin who worked with Watson and Crick has only been recently acknowledged. Why is the role of women frequently overlooked? Are they hiding in plain sight? Are we at fault for not applauding our fellow women’s contributions more loudly? This article will examine the evidence of women’s roles in imaging over the last 100 years. As Dr Beverley Coleman said during her acceptance speech of the Gold Medal from the Radiological Society of North America (RSNA) in 2024—“I should not be standing here…”—she is the first African American woman to be given the gold medal and she shared with us her journey.^1^ She commented on the cultural difficulties she had encountered during her working life, but even with the support from her colleagues and her family, she was still surprised at how much she had achieved.

There is some evidence that over the past 20 years the role of women in leadership positions has improved significantly compared to the previous 100 years. Leadership roles such as presidents of the Royal Colleges,^2^ chairmen of the boards of the international societies,^3^ gold medallists^1^^,^^4^ and honorary memberships/fellowships, and senior editors of the journals are publicly available. The number of females in these roles compared to men and the change in distribution over time can give us an indication of whether we are moving in the right direction. Publicly available information is a good starting point to look at gender balance over this past century.

Since the Royal College of Radiologists (RCR) was formed in the United Kingdom in 1975, there have only been 5/18 (28%) female presidents. However, since the first female president (Professor Dame Janet Husband) was elected in 2004, the majority (5/7) have been women.^2^ The largest radiological society in the world with over 140 000 members from 182 countries is the European Society of Radiology (ESR). Since the Board was formed in 2005, the number of female presidents/chairmen was only 6/21 (28%) but the majority have been female in recent years. As the chairman is drawn from the Board it is encouraging that in 2025 more than half of the Board members and sub-committee chairs are female (based on research undertaken by the European Society of Radiology, 2025). Radiological Society of North America has had a similar percentage of female presidents since 2000 (7/24 (29%)) to the other large societies but with no increasing trend, and with the 2024 Board still only having 7 female members (30%). However, the RSNA record has improved compared to the previous 100 years when there was only 1 female president in 1995 and 2 female board members since RSNA was formed in 1918.^3^

Gold medals are awarded by the RSNA Board for exceptional contribution. The first woman to receive this was Dr Maud Slye in 1922, who bred mice to demonstrate that cancer was a recessive disease that could be eradicated by mate selection. Two years later, Marie Curie was recognized for her extraordinary contributions, followed by Edith Hinkley Quimby for her work in nuclear medicine and radiation physics.. Dr Lucy Frank Squire was recognized for her contribution to teaching and Alice Ettinger, originally from Berlin, for bringing spot radiography to Boston, USA, together with modern gastroenterological techniques. Adele Swenson was awarded one in 1984, followed by Eleanor Montague for establishing breast-conserving therapy and improved radiation therapy techniques, and then Rosalyn Sussman Yalow, a medical physicist who developed the radioimmunoassay. These 8 women represent 5% of the recipients between 1919 and 1999. However, since then, 21% of gold medals have been awarded to women.^1^

Similarly, the Gold Medal of the American College of Radiology was awarded to only 18 women since it was founded in 1920 but from 2000 there have been 10 female recipients, increasing the rate to 14%.^4^

## Why is it important to have women in leadership roles?

It is widely acknowledged that women bring a sense of balance and calming, thoughtful influence to organizations. When women are working in a group on cognitive tasks, they enhance the general collective intelligence of the group resulting in improved decision-making.^5^ Women’s ability to multitask is legendary. One study demonstrated that they are better at rapidly switching between tasks whereas more men struggled to cope with juggling priorities. Stoet et al. concluded that women were more organized under pressure and spent more time planning in a stressed, complex situation than men. On average, women were able to switch more easily between tasks than men.^6^ There is no evidence that women have better time management skills than their male counterparts. However, successful women demonstrate that time management skills are an absolute necessity as they juggle career and caring or family responsibilities. They often achieve this by careful planning of their working day, effectively prioritizing to do lists and trying to complete tasks before embarking on the next activity—often challenging when there are many competing demands on their time.

Women leaders are needed as role models to inspire the upcoming generation of female radiologists. Gonzalez-Perez et al. evaluated female role model intervention in the STEM job sector^7^ where female volunteers working in STEM subjects went into schools to talk to girls about their careers. They showed an increased interest in mathematics, and expectations of success in STEM subjects. Female role models are particularly important whether there is a perceived gender bias, institutional barriers or negative stereotypes. The specialty of radiology has less perceived barriers for women compared, for example, to surgery.

Studies have shown that women increase productivity, enhance collaboration and organizational performance, improve fairness, and promote inclusive workplace cultures. Women tend to show high levels of compassion, demonstrate a more empathetic leadership style and have better team-building skills. In a survey of 96 000 employee-year observations from 2017 to 2019 examining the impact of female managers on well-being, occupational stress, job satisfaction, and workplace cohesiveness, the findings supported the transformational leadership behaviours. Women managers improved workplace engagement and decreased work-related stress.^8^ The excellent article by Jean Seely highlights reasons why women enhance the workplace.^9^ She shows that women improve patient-centred care and communication, foster innovation, and bring unique insights and collaboration when tackling challenges.

## What needs to change?

Spalluto et al. identified key steps that leaders can take to increase and retain women in radiology: (1) marketing radiology to talented female medical students, (2) addressing recruitment and bias to ensure women are selected in equal numbers, (3) understanding and accommodating the provisions of the Family and Medical Leave Act of 1993 and the Fair Labor Standards Act for both trainees and radiologists in practice, (4) preventing burnout and promoting well-being, (5) offering flexible work opportunities, (6) providing mentorship and career advancement opportunities, and (7) ensuring equity with regard to conditions of employment and pay.^10^ Strategies for thriving in radiology include ensuring uninterrupted reporting time, efficient work practices, transparent work policies for parental leave, sick leave, childcare, time to strategize and optimise workflow, flexible work patterns with hybrid work patterns and supportive colleagues.^9^

We need women to want to take on a leading role. Describing the glass ceiling in academic radiation oncology, Corrinne Faivre–Finn makes a plea for women to volunteer for leadership positions.^11^ In 2017, there were few female academics despite almost 30% of females joining American training programmes.^12^ By 2020, only 17% women held senior positions in radiation oncology, and only 12% were academic chairs.^13^ The American Psychological Association noted that identifying potential leaders early, establishing mentorship and sponsorship programmes, supporting women to join women-led professional organizations, and focussing on allyship all helped to improve the chances of younger women becoming leaders of the future. The Women in Radiology (WiR) initiative has been very effective in increasing the number of women in academic positions and in radiology generally. The American Association of Women in Radiology (AAWR) has created a toolkit providing information about collaborating, initial planning, recruiting members, determining the mission of the group, and acquiring institutional support and sponsorship.^14^

## The importance of equality, diversity, and inclusivity

Following the civil rights movement in the United States, the “Equality, Diversity and Inclusion” initiatives started in the mid-1960s. This led to workplace diversity policies and the legal framework for affirmative action mainly through the executive orders from the US Presidents Kennedy and Johnson. There were societal movements and legal changes that began to influence big corporations. Diversity training was introduced into the workplace with courses on unconscious bias for interviewers on selection panels. Affirmative actions include all-female shortlists and designing job advertisements to attract ethnicities or genders. The UK Equality Act 2010 supports gender-balanced selection panels and shortlisting. This is difficult if there are insufficient women or ethnicities coming through the training pipeline. However, many medical schools now take equal or greater numbers of women compared to men. Larger numbers of women are choosing radiology as a career which increases the pool of talented individuals from which to promote to senior positions.

Since the Covid19 pandemic, economic pressures have meant that the corporate world is less likely to fulfil their EDI obligations and focus instead on returning value to their shareholders. In academia, this pressure is less apparent, and EDI is still valued but perhaps less so in hospitals and clinical practice. Recently, the US government created an executive order to remove the need for diversity policies. This may not be in the best interests of women or the ethnic minorities. There is no question that the best, most qualified person should be appointed but the EDI initiative resulted in more effort to find the best women as well as the more obvious men and finding excellent individuals from all ethnicities. It is important not to lose sight of this.

## Where are we with the workforce gender bias?

In order for there to be more female leaders, there needs to be sufficient women working in radiology. In the United Kingdom, in 2023, 36% of consultant clinical radiologists were female with only 12% of women specializing as interventional radiologists. At the level below this, 56% Specialist and Associate Specialist consultants (a doctor who has the same clinical experience as a consultant but who has not completed specialist training) are female.^15^ Specialist and Associate Specialist positions are given to individuals who trained out with the United Kingdom.

A report in 2019 found that in most European countries substantially less than half the radiology workforce was female despite parity at medical school level.^16^ Overall, 30% of the ESR membership was female although this is higher at 41% in the younger age groups. The data from 2024 (Table 1) show a marked improvement with 41.2% overall being female with 49.8% in the youngest age group. These data begin to explain the low number of women in senior positions in previous decades but point to a more promising, equitable future.

**Table 1.** Overview of the composition of the European Society of Radiology (ESR) membership in 2024, stratified by gender and generation (based on research undertaken by the European Society of Radiology, 2025).

	Born before 1946	Baby boomers Born 1946-1954 Aged 70-79	Baby boomers II Born 1955-1964 Aged 60-69	Generation X Born 1965-1979 Aged 45-59	Gen Y millennials Born 1980-1994 Aged 30-44	Generation Z Born 1995-2010 Aged 14-29	All generations
Total ESR members 2024	1271 (0.9%)	4672 (3.3%)	14 886 (10.4%)	41 932 (29.4%)	66 663 (46.8%)	13 043 (9.2%)	142 467 (100%)
Male members	1100 (87%)	3704 (79%)	10 434 (70%)	25 560 (61%)	36 270 (54.4%)	6526 (50.2%)	83 594 (58.7%)
Female members	171 (13%)	968 (21%)	4448 (29.9%)	16 327 (38.9%)	30 335 (45.5%)	6493 (49.8%)	58 742 (41.2%)
Diverse members	–	–	4 (0.1%)	45 (0.1%)	58 (0.1%)	24 (0.2%)	131 (0.1%)

**Figure 1.**
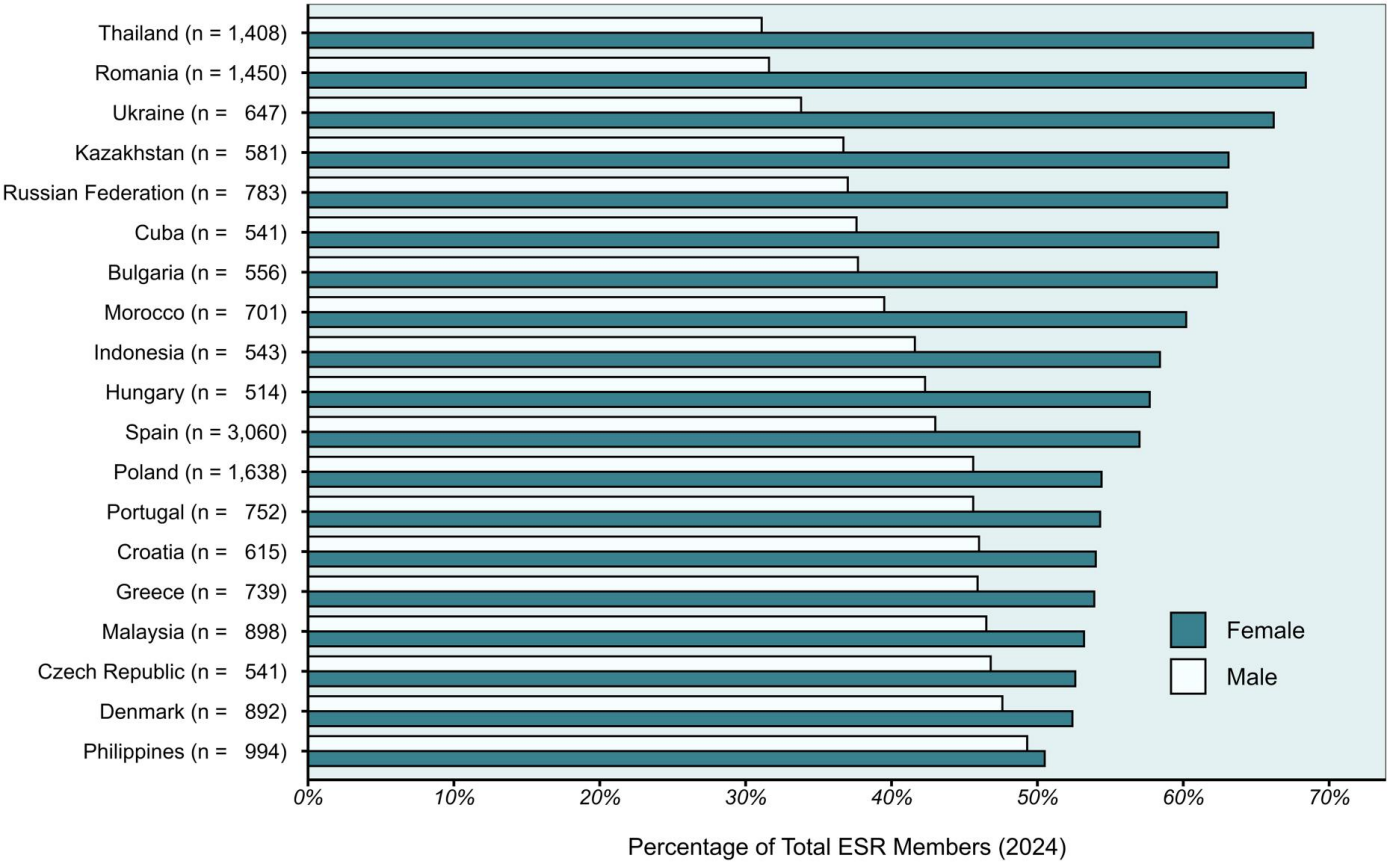
Male and female percentages in countries with over 500 radiology members (based on research undertaken by the European Society of Radiology, 2025).

**Figure 2.**
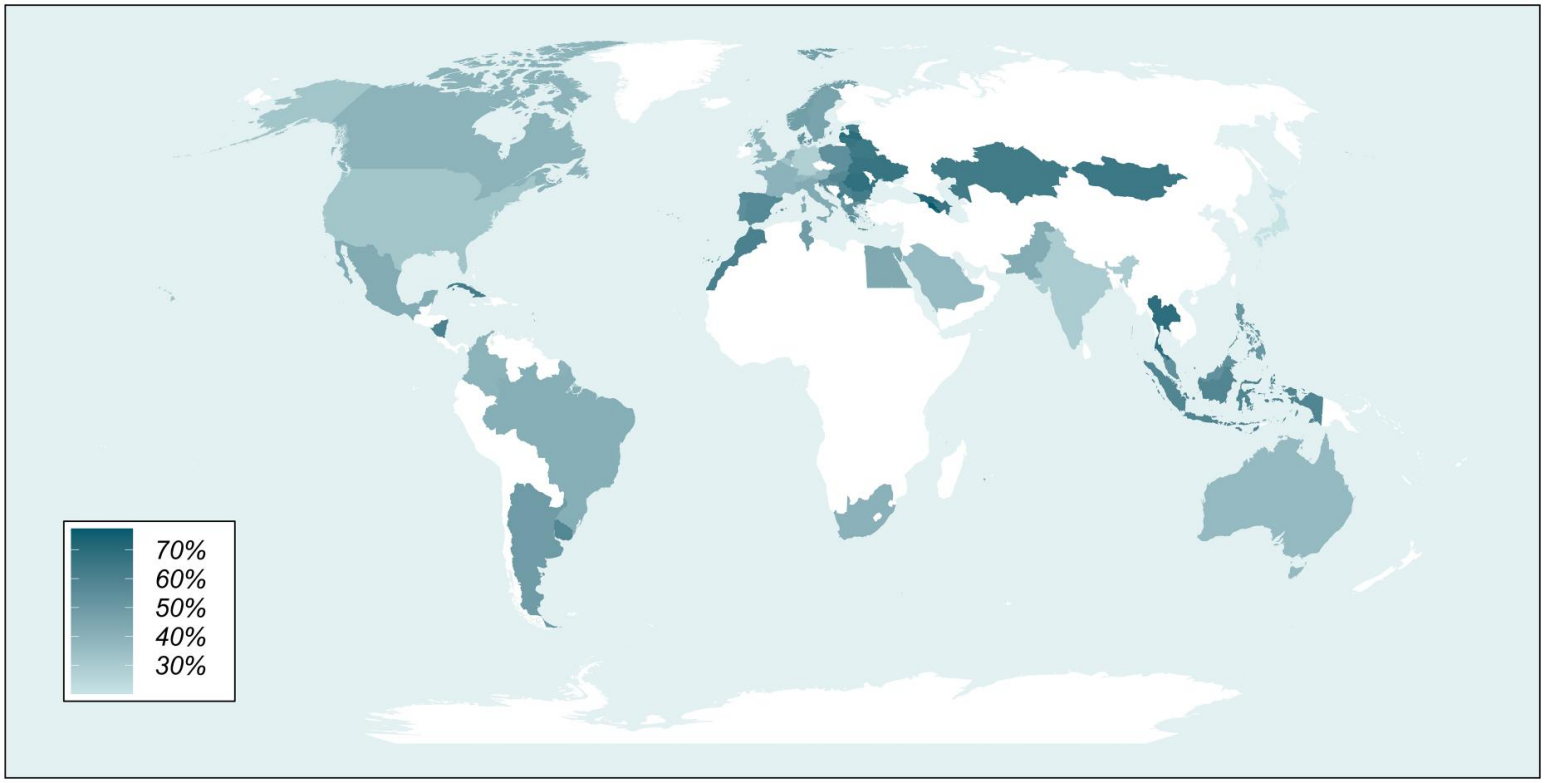
World heatmap representing the percentage of female ESR members in each country (based on research undertaken by the European Society of Radiology, 2025).

When we examine the female-to-male ratio in different countries, there are marked differences. A 2018 survey of 26 countries societies revealed that overall, 33.5% of radiologists were female, with the US having the lowest proportion at 27.2% and Romania, Spain, and Thailand having over 50% women.^17^ The ESR male-to-female ratio is a reasonably reliable barometer as most international radiology societies offer free membership of the ESR as part of their membership package. The 2024 data from ESR show that the countries with the highest ratio of female-to-male members between 66% and 77% are Armenia, Moldova, Georgia, Latvia, Thailand, Romania, Lithuania, and Ukraine (Table 2) and Figure 1 shows a graph of male-to-female ratios in countries with over 500 members. Looking at countries with more than 1000 ESR members, Spain and Poland have 57% and 54.4% female members, respectively, while those countries with less than 33% female members are USA, India, Germany, Venezuela, and Japan. Perhaps the most striking way of illustrating the disparities is on the map of the world (Figure 2).

**Table 2.** Countries with more than 50% female radiologists as members (based on research undertaken by the European Society of Radiology, 2025).

Country ▼	Female (%)	Male (%)	Total ESR members 2024
**Armenia**	77.3	22.0	132
**Moldova, Republic of**	76.2	23.4	231
**Georgia**	71.1	28.6	312
**Latvia**	69.3	30.4	352
**Thailand**	68.9	31.1	1408
**Romania**	68.4	31.6	1450
**Lithuania**	66.3	33.7	354
**Ukraine**	66.2	33.8	647
**Azerbaijan**	65.0	35.0	143
**Mongolia**	64.7	35.3	138
**Montenegro**	64.7	35.3	119
**Belarus**	64.4	35.6	119
**Kazakhstan**	63.1	36.7	581
**Russian Federation**	63.0	37.0	783
**Estonia**	62.8	37.2	290
**Cuba**	62.4	37.6	541
**Bulgaria**	62.3	37.7	556
**Morocco**	60.2	39.5	701
**Nicaragua**	60.0	39.2	130
**Serbia**	59.5	40.5	351
**Bosnia and Herzegovina**	58.8	40.8	250
**Indonesia**	58.4	41.6	543
**Hungary**	57.7	42.3	514
**Uruguay**	57.7	42.3	260
**Spain**	57.0	43.0	3060
**Slovenia**	55.1	44.7	421
**Poland**	54.4	45.6	638
**Portugal**	54.3	45.6	752
**Croatia**	54.0	46.0	615
**Greece**	53.9	45.9	739
**Slovakia**	53.8	46.2	239

When we examine the number of women in departmental chair positions internationally, it is not only a reflection of the percentage of women in the “pipeline.” An ACR workforce commission showed that only 7% of women, compared to 14% of men, were in leadership position.^18^ The 2015 report of major US academic departments showed that only 9% of chairs were women despite 34% of academics being female.^19^ In Spain, despite the high proportion of female radiologists only 42.9% were section chiefs and 24.4% departmental chairs.^20^ In Italy, where half the workforce are women, only 11% are professors and 14% departmental chairs.^21^ In Japan, only 1% are female chairs, with Germany having a similar small number. Overall, the ESR found 20.6% of department chairs were female. If it was only a pipeline problem, then countries with high numbers of female radiologists should have greater numbers of senior women but this does not seem to follow.

Authorship of publications can give a guide as to who is contributing to the specialty, with first and last positions giving an idea of seniority. When we look at the number of first and last authors on academic publications in the ESR family of journals, the numbers of women are 34.6% and 25.8%, respectively, in papers published in 2022-2024. Looking at presenters of abstracts and speakers in the educational programme of the annual European Congress of Radiology, the number of females is higher at 44.5% and 37.4%, respectively. This could reflect the pipeline of younger academics coming through and if so, is more encouraging.

Editorial positions of major journals are regarded as influential leadership roles. *Radiology* has had a female editor from 2023 to 2025, and two-thirds of the main editorial board are also women. Similarly, there are female editors of *Clinical Imaging*, *Radiographics*, and *Journal of the American College of Radiology.*^22^ Across the entire BIR journal portfolio, there are 41% of female editors across all editor roles, e.g. Editors-in-Chief, Senior Editors and Associate Editors. This has been a considerable improvement since 2016 when most of the Board were white male from the United Kingdom. Other positions of influence, for example, the ECR programme planning committee, the subcommittee chairs and the subcommittee have equal numbers of men and women. This has been the situation for the last 4 years. In the European Society of Breast Imaging, we have been fortunate to have equal numbers of men and women across Europe willing to serve on committees, but this is partly due to the high numbers of female breast imagers.

## Conclusion

Current data show that women are still underrepresented in senior leadership positions in radiology. The proportion of females in these positions has been improving over the past 2 decades. This is partly due to the increased numbers of females in medical schools and in the number of women choosing to become radiologists. Women wish to be promoted on quality and excellence rather than as part of a quota, but they require encouragement and inspiration to take on additional responsibilities. The benefit of a more diverse community contributing to decision-making and steering the direction of radiology is not in question. There should be an active pull of women into committees in recognition of the enormous and different contribution they make. Often this means one must look a little harder to find the excellent women who work with us, give them an opportunity and support them. They are often hiding in plain sight.
